# Quantifying thermal cues that initiate mass emigrations in juvenile white sharks

**DOI:** 10.1038/s41598-022-24377-1

**Published:** 2022-11-18

**Authors:** Emily Spurgeon, James M. Anderson, Yi Liu, Vianey Leos Barajas, Christopher G. Lowe

**Affiliations:** 1 Department of Biological Sciences, California State University, Long Beach, 1250 Bellflower Blvd, Long Beach, CA 90840 USA; 2 School of Environmental Science and Engineering, Southern University of Science and Technology, Shenzhen, China; 3 Department of Statistical Science and School of the Environment, University of Toronto, 27 King’s College Cir, Toronto, ON M5S 1A1 Canada

**Keywords:** Marine biology, Animal migration, Behavioural ecology

## Abstract

While the function of migration varies among species, environmental temperature is known to be one of the most important abiotic variables that drive animal migration; however, quantifying the thresholds and timing of the cues that influence a mass emigration is difficult, often due to lack of monitoring resolution, particularly for large, highly mobile species. We used acoustic telemetry tracking and high-resolution water temperature data over a relatively large spatial scale (5.5 km^2^) to identify and quantify a thermal threshold for mass emigration of juvenile white sharks. Sixteen tagged sharks were observed to initiate a search for warmer water within 10–12 hours of an upwelling event where water temperatures dropped below 14 °C. Eleven sharks traveled ~ 35 km away where they experienced similar cold temperatures before returning to the aggregation site within 24 hours. Five days following the upwelling event, most sharks emigrated from the site for the season. Quantifying movement patterns across different spatial and temporal scales is necessary to understand cues and thresholds influencing animal migration, which may be greatly affected by climate anomalies and climate change, resulting in potential impacts on the dynamics of local prey species, management, and conservation policy and practice.

## Introduction

The distributions and migratory patterns of both terrestrial and aquatic animals are seasonably variable^1,2^. The motivation for animals to relocate can be driven by both abiotic and biotic influences and may differ between the sex and ontogenetic stage of the species^2–5^. Some animals move out of the need to find a more physiologically suitable location (e.g., warmer, more humid, etc.)^6^, while others relocate to follow a food source^7^ or to find a suitable place to breed or raise their young^8^. However, regardless of motivation, relocation often occurs in response to environmental cues.

Ambient temperature change is a key variable in driving animal migration for many species, particularly ectotherms^9–14^. Thus, animals often seek out and use habitats within their thermal preferenda, where they may be most energetically efficient^15–20^. For many temperate marine species, there can be short-term, broad changes in temperature within an occupied habitat that temporarily exceed the animal’s thermal preferenda; however, individuals may tolerate the temporary physiological costs of a greater fluctuation in temperature given adequate protection and food availability^21^. Although these species may have a wider thermal tolerance range than more stenothermic species, there is still a threshold where the physiological cost of remaining within suboptimal temperatures outweighs the benefits provided by the area it is occupying. When this threshold temperature is exceeded, an individual should seek out more favorable conditions, thereby initiating a migration. Quantifying this threshold requires information about the magnitude, duration, and spatial extent of these thermal changes that would induce a synchronized mass emigration^22–25^.

White sharks (*Carcharodon carcharias*) are known to seasonally migrate. The drivers of migration are ontogenetically variable—e.g., adults may migrate in response to the movement of key prey species^26^, whereas juvenile migrations are likely influenced by seasonal changes in environmental conditions^27–29^. Although adult white sharks exhibit regional endothermy^30^, young-of-the-year (YOY) and small juvenile white sharks (JWS) likely lack sufficient body mass and thermal inertia needed to efficiently maintain a warmer body temperature and therefore may be more temperature sensitive^15,19,27,31–33^. White sharks are one of the only regional endothermic fishes that utilize nearshore nursery habitats, which suggests that JWS may rely on behavioral thermoregulation by inhabiting warmer water than adults^19,33–36^. However, little is known regarding the thresholds and magnitude change of these thermal cues for JWS, and how they may influence the decision to remain at, or leave, a habitat resource.

The Southern California Bight (SCB), extending from Point Conception, California (United States) to Cabo Banda, Baja California (Mexico), is an example of a temperate marine environment home to the nursery habitats for the northeastern Pacific populations of white sharks^27,34,36^. These nursery areas are commonly found in shallow (< 200 m deep) and nearshore (< 20 km offshore) waters where juvenile white sharks form loose aggregations at “hotspot” locations and display a high degree of residency^20,27,33,34,36,37^. JWS form these aggregations at specific locations using relatively small, nearshore areas (< 8 km^2^)^38^ with similar conditions (i.e., thermal profile, habitat composition and structure, etc.). Previous data have documented that the highest relative abundance of JWS distributed along the SCB coastline (< 500 m from shoreline) occurred in the summer and fall months when the water is at its warmest of the annual cycle^20,25,27^. During colder winter months when the water temperature fell below 14 °C for an extended period, young-of-the-year (YOY) exhibited a large-scale southward migration (> 500 km) into the Mexican waters of Baja California where winter temperatures were similar to those of Southern California summer months^26,27,36^. Anecdotal evidence suggests these migratory patterns appear to be changing and more individuals are staying in southern California throughout the entire year. Changing thermal conditions may be the cause of this disruption to long-established seasonal migration behaviors across all size classes of JWS. Increased variation of seasonal water temperature makes understanding the drivers of seasonal migration an invaluable tool in the conservation efforts of the species, particularly because fishing mortality rates are markedly higher in Mexican waters than in California^39^. It is therefore vital to understand not just the historical patterns of JWS movement, but the environmental cues they use to make their decisions, so we may better anticipate what to expect under changing climate scenarios.

We used detection data from a high-density acoustic receiver array to track the movements of 25 individual JWS that were resident within a Southern California nursery hot spot between 15 May 2020 and 10 December 2020, with respect to the environmental conditions present. Our study quantified the specific temperature thresholds that triggered a mass emigration in these JWS from their long-term aggregation site as well as the response time of the JWS through behavioral analysis of residency patterns at an aggregation location, and through the synthesis of high-resolution movement and temperature data gathered in situ. Since site-specific aggregation behavior of predators can have top-down effects on community structure, understanding the link between environmental temperatures, and the propensity to “stay or go” in JWS will help determine reveal how any changes in seasonal residency and habitat use patterns will impact the wider ecosystem.

## Materials and methods

### Study site

In 2020, an aggregation of JWS was monitored using passive acoustic telemetry near Carpinteria, CA at Padaro Beach to better understand habitat use and their fidelity to the area. This location constitutes a typical nearshore and shallow JWS nursery habitat. Padaro Beach is characterized as a sandy beach with a rocky reef adjacent to an estuary inlet and is considered low wave energy compared to many of the other nursery habitats available. Water depths in this area range from 0 to 10 m, at distances of 5 m from the shoreline to ~ 1000 m offshore. Tagged individuals were observed across approximately a 5.5 km stretch of coastline.

### Tagging

We tagged 25 individual JWS at the study site between 3 April and 4 September 2020. All individuals were externally dart-tagged with either Innovasea V16 coded acoustic transmitters (n = 9, V16-4x, V16-6x, 152–158 dB, 4-year battery life), Innovasea V13 coded acoustic transmitters (n = 10, V13-1x, 147–152 dB, 2-year battery life), or Innovasea V13P coded acoustic transmitters (n = 6, V13P-1x, 147–152 dB, 1-year battery life) equipped with a pressure sensor (0.15 m resolution, 34 m depth rating). All transmitters had a pulse interval between 40 and 180 s to allow at least hourly detectability regardless of tag density. A camera at the end of a 5 m long pole was submerged under the shark to identify sex before tagging. The approximate size of the individual was determined using aerial drone video analysis at the time of tagging. Length estimates for the JWS ranged from 153 cm total length (TL) to 315 cm TL with size classes broken down as follows: young-of-the-year (YOY) sharks between 151 and 175 cm TL (n = 4), larger juveniles between 176 and 300 cm TL (n = 19), and subadult between 300 and 315 cm TL (n = 2)^36^.

### Acoustic monitoring

**Figure 1:**
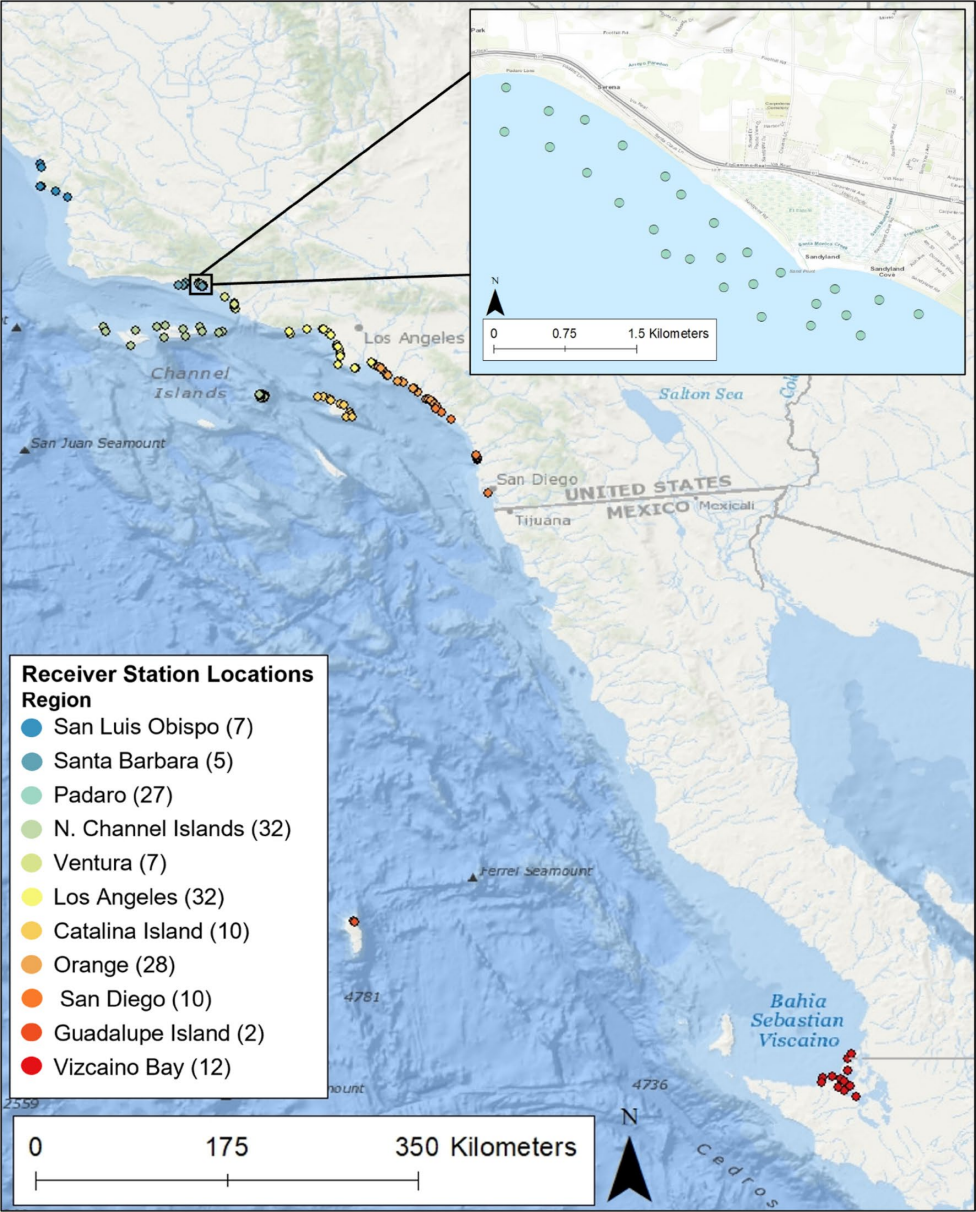
The locations of acoustic receivers along the California and Mexican coastline. Each color represents a region where (n) receivers are located. The inset map shows the higher density receiver array at Padaro Beach, CA. Map was produced using ArcMap™ version 10.8.1 with the “Ocean” basemap. https://www.arcgis.com/home/item.html?id=5ae9e138a17842688b0b79283a4353f6.

A grid array of 27 omni-directional VR2W (n = 10) and VR2Tx (n = 17) acoustic receivers spaced 250–500 m apart were deployed throughout the ~ 5.5 km^2^ study site to maximize the detection probability of any tagged JWS in the area between 15 May and 9 December 2020 (Fig. 1). Receivers were moored < 1 m from the seafloor in water depths ranging from 2.7 to 9 m. In addition to monitoring JWS presence within this aggregation site, a network of 145 VR2W acoustic receivers dispersed across the region (Southern California Acoustic Telemetry Tracking Network—SCATTN), was used to monitor the large-scale movements of the JWS throughout the SCB extending up to Morro Bay, CA to track where the JWS moved once they left their aggregation site (Fig. 1).

### Ethics statement

All tagging and tracking procedures were approved and conducted under the guidelines of the California State University Long Beach Institutional Animal Care and Use Committee (IACUC); protocol #364. This study is reported in accordance with all ARRIVE 2.0 guidelines.

### Environmental monitoring

To measure how temperature influences JWS movement away from the aggregation site, we measured seafloor and surface water temperature (SFT and SST) changes across the array at each of the 27 receiver stations. Hourly temperature was monitored ~ 1 m from the seafloor using either HOBO Stowaway TidbiT v2 UTBI-001 (n = 9, 0.1 °C resolution and ± 1 °C accuracy), the internal temperature logger of the VR2Tx receivers (n = 17, 0.1 °C resolution with ± 0.5 °C accuracy), or an aquaMeasure sensor (n = 1, 0.01 °C resolution, ± 0.2 °C accuracy). Sea surface temperature was collected in the first 0.5 m of water using either Electric Blue EnvLogger (n = 3, 0.1 °C resolution and < 0.2 °C accuracy) or the built-in temperature sensor on the Rx-Live acoustic receiver (n = 1, 0.01 °C resolution, ±0.2 °C accuracy) placed on four different receiver station locations. Sea floor water temperature was also monitored at a majority of the 145 receiver stations as part of the SCATTN array.

### Data analysis

To eliminate false positive detections, valid detections of tagged animals were filtered from our database. A shark was considered a unique individual present if it was detected by any receiver within the array at least twice each day. We averaged all unique individuals detected within a given hour or day and calculated the proportion of individuals present relative to the number tagged. The only sharks included in this analysis were sharks that were tagged and detected at this aggregation site (Padaro Beach). Average SFT and average SST and their standard deviations were also calculated for both hourly and daily time resolutions across all 27 receiver stations. The dependent variable (shark presence), and the independent variable (temperature), were aligned according to their times to determine if there were correlations between shark presence and the environmental temperatures (e.g., SFT, SST, ΔT). All data were filtered and organized using R version 1.3 unless otherwise stated^40^.

#### Cross-correlation function and lagged regression

To account for any delay in animal response to dramatic changes in temperature, we used the *acf* (Auto- and Cross- Covariance and -Correlation Function Estimation) function in R, which is a cross-correlation function, to transform the data to discern any relationships between environmental temperature and shark presence^41^. The time series daily average SFT dataset is defined by x_t_ and the number of unique sharks present in the aggregation site is defined by y_t_, the cross-correlation function defines a set of correlations between x_t+h_ and y_t_ with t being time and h being the time shift (e.g., one day). If the highest correlation values occurred when h was negative that meant the temperature at a time before t_0_ led shark presence at t_0_, with t_0_ representing the time with no shift (e.g., h = 0). In this case temperature changed first and then shark presence had a delay in response. However, shark presence may be a function of either temperature at a previous time, or it may be a function of the shark presence at a previous time.

To statistically determine if shark presence (y_t_) is a function of temperature at any given previous time (*x*_*t*+*h*_), a generalized linear model (GLM) was used to compare the number of unique sharks present against both various temperature lags as well as previous shark presence. Previous shark presence was included to show if temperature is influencing shark presence, or if the JWS are just there because they were there the day before. Several combinations were run comparing the number of unique sharks present with different combinations of shark and temperature lag. The model with the lowest Akaike Information Criterion (AIC) values was chosen and if several models had AIC values within 2 points from one another, the residuals from each model were visually inspected to choose the final model.

#### Change point analysis

We used the pruned exact linear time (PELT) method^42^ to detect the turning points in time series of mean values of sea temperature and both the proportion of tagged sharks present as well as the total unique sharks present. This method is a wrapped function named *findchangepts* in Matlab. We set a maximum of two change points for the analysis from 15 May to 9 December to represent the entire monitoring period and one change point for the analysis from 4 September to 9 December and from 1 to 16 November to capture the times once all sharks had been tagged. When setting maximum change points to be K, the algorithm will calculate the total residual errors (J) for each possible change points model with change points no more than K (Eqs. 1, 2 and 3). That means even if we set two maximum change points, the function will only return one change point if the added change point does not decrease the total residual errors. This change point analysis was conducted on both the hourly and daily resolution datasets.

1 $$mean\left(\left[{x}_{m}\cdots {x}_{n}\right]\right)=\frac{1}{n-m+1}\sum_{r=m}^{n}{x}_{r}$$

2 $$TotalVar\left(\left[{x}_{m}\cdots {x}_{n}\right]\right)=\sum_{i=m}^{n}{\left({x}_{i}-mean\left(\left[{x}_{m}\cdots {x}_{n}\right]\right)\right)}^{2}$$

3 $$J\left(K\right)=\sum_{r=0}^{K}TotalVar\left(\left[{x}_{{k}_{r}}\cdots {x}_{{k}_{r+1}-1}\right]\right)$$

${k}_{r}$ is the index of the rth change point. ${x}_{{k}_{0}}$ and ${x}_{{k}_{K+1}-1}$ are defined as the first and the last sample of the signal.

## Results

**Figure 2:**
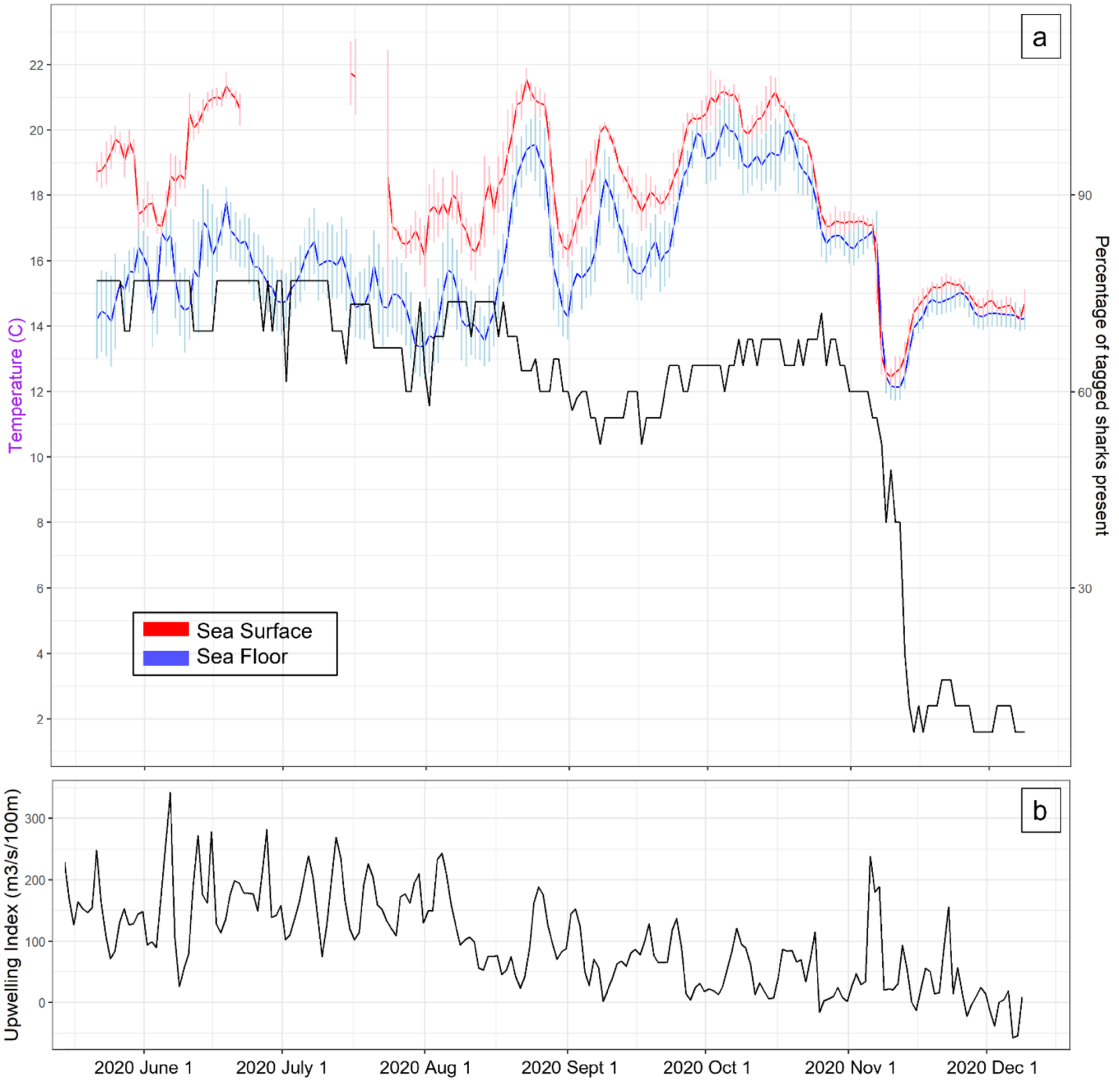
(**a**) Sea surface and sea floor temperature plotted with the proportion of tagged sharks (solid black line) that were detected at Padaro between 15 May and 10 December. The error bars around the seafloor and sea surface temperatures represent the 95% confidence interval. (**b**) Bakun Upwelling Index Values of daily average wind-driven cross-shore transports for the region of 33 N, 119 W. Positive numbers represent upwelling and negative numbers represent downwelling.

**Figure 3:**
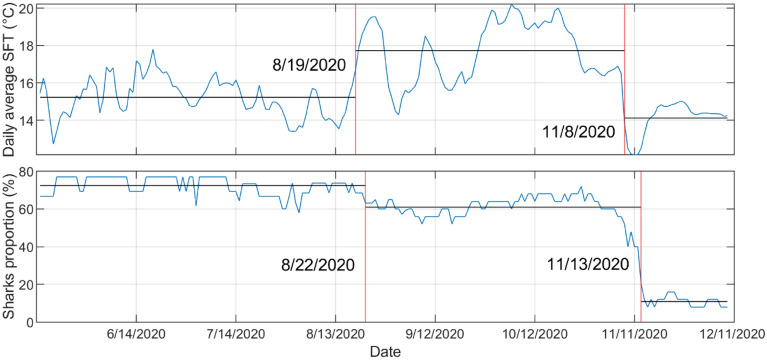
Change point analysis of daily averaged sea floor temperature data and proportion of sharks present at Padaro from 15 May to 10 December. Two change points were detected for both temperature and shark presence, the first on the 19 and 22 of August and the other on the 8 and 13 of November as represented by the red solid lines.

**Figure 4:**
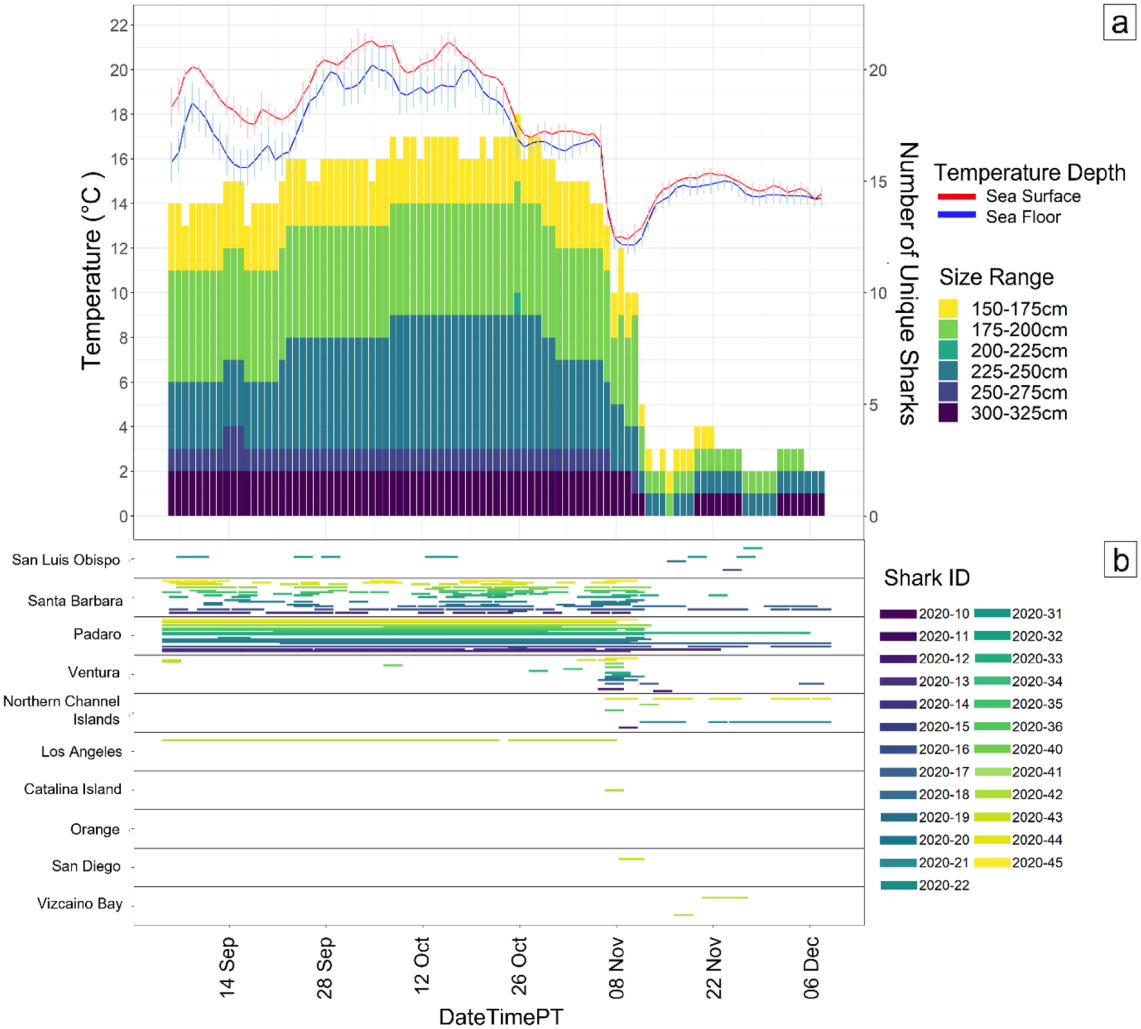
From 4 September to 10 December (**a**) shows a stacked bar plot of different size classes of JWS and how many unique sharks were present each day at Padaro Beach plotted with the sea surface and sea floor average daily temperatures. (**b**) Abacus plot of what regions the unique sharks that were detected at Padaro were present in. Regions are ordered by latitude.

**Figure 5:**
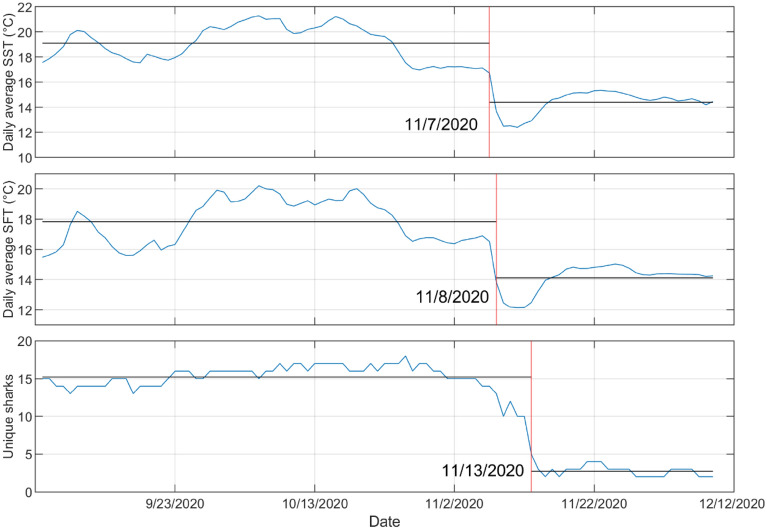
Change point analysis from daily averaged data from 4 September to 10 December of sea surface temperature, sea floor temperature, and the number of unique sharks present. The change points occurred on 7, 8, and 13 November, respectively represented by the solid red lines.

Throughout the study period from 15 May to 9 December 2020, there were over 3.8 million detections of tagged JWS in the monitored aggregation site from 25 unique individuals, across the 27-receiver array. The water temperature significantly fluctuated during that time with SFT ranging from 10.9 to 23.8 °C (daily averages 12.1 °C–20.2 °C) and SST ranging from 12.1 to 25.2 °C (daily averages 12.4 to 21.5 °C) (Fig. 2a). From May to December there were two notable change points relative to temperature changes where tagged sharks initiated movements away from the aggregation hot spot (Fig. 3). The first temperature change occurred on 19 August, when the water began to mix, breaking down the thermocline and creating increasing warmer temperatures throughout the water column. Approximately three days later on 23 August, 15% of the tagged sharks present left the area in response (Figs. 2a and 3). The second notable change occurred in November, when the daily average SST and SFT dropped 2.7 °C on 8 November due to a strong upwelling event in the region and dropped 4.4 °C over four days. Following the peak of this upwelling event, over the course of five days, 87% of the tagged sharks present at the aggregation site left (Figs. 2a and 3). Upwelling was determined using the Bakun Index Values from NOAA/NMFS/PFEG from the region encompassing the aggregation site (33 N 119 W) (Fig. 2b)^43^. During these five days when the sharks began to leave, if they were not detected at Padaro Beach, they were primarily detected in neighboring regions (< 35 km). By 13 November individuals began to disperse throughout the SCB, four moving north up to San Luis Obispo County (> 210 km) and two moving into Baja Mexico waters (> 1000 km) (Fig. 4). This was observed across sharks of all size classes. There was minimal difference between the average daily changepoints of sea surface and sea floor temperatures (Fig. 5), indicating the disappearance or lack of a thermocline within the nursery aggregation hot spot.

The cross-correlation function performed on the daily temperature-shark presence dataset showed the highest correlation between the number of unique sharks present and temperature indicating a 5-day lag in response. Several GLM models were run with different combinations of shark presence or temperature lags to statistically confirm this lag correlation and two models were within two AIC points of each other. We visually inspected the residuals of the two models to choose the model of best fit. The best fit GLM confirmed that while temperature lag of three, four and six days were significant (*p* = 0.03, p = 0.04, and *p* = 0.03), temperature lag of five days showed the strongest correlation (*p* = 0.003). The model also showed that the presence of sharks the day before (t_-1_) was also highly correlated to the presence of sharks at t_0_ (*p* = 5.28e−09).

**Figure 6:**
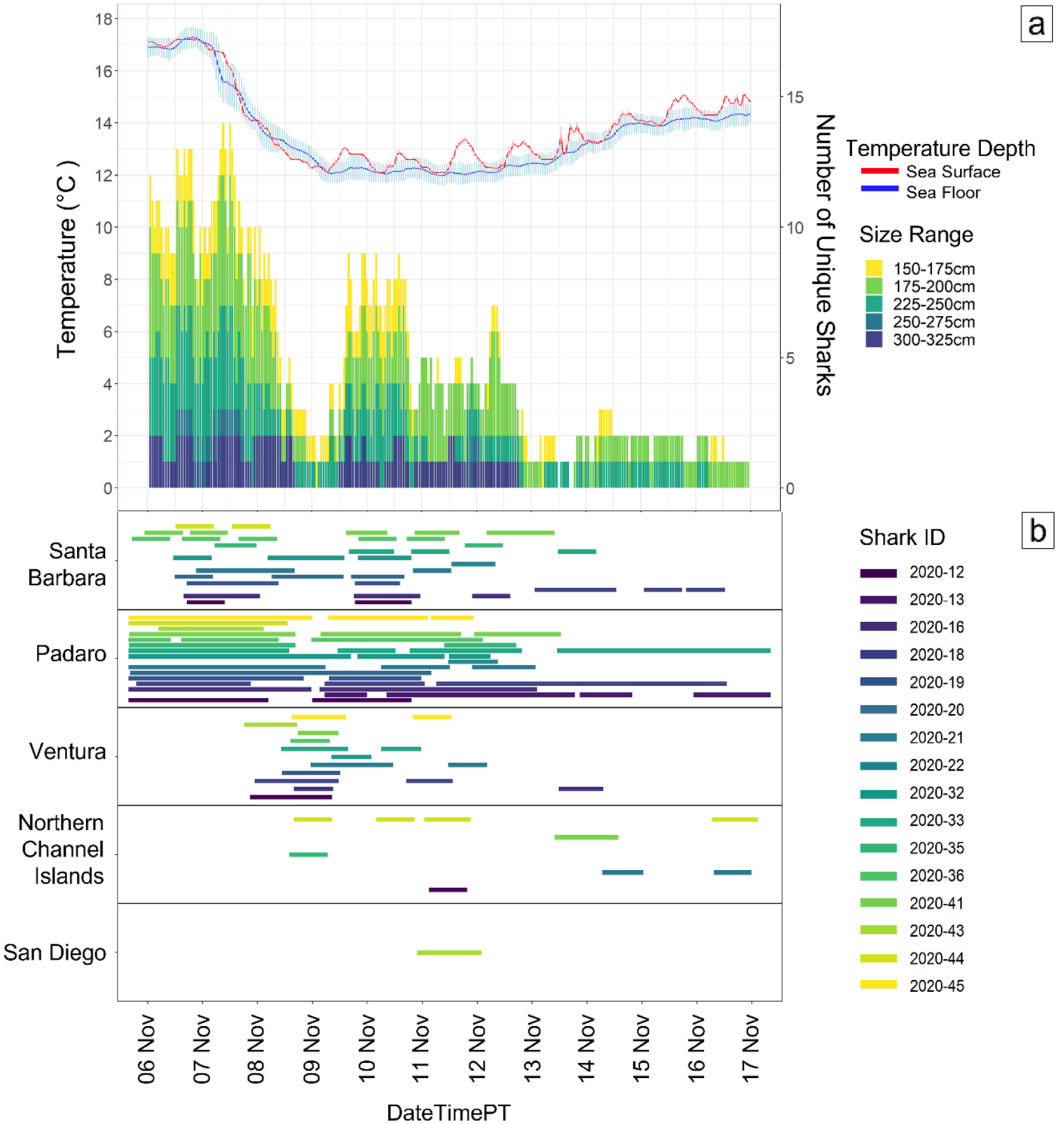
From 6 to 16 November (**a**) shows a stacked bar plot of different size classes of JWS and how many unique sharks were present each hour at Padaro Beach plotted with the sea surface and sea floor average hourly temperatures. (**b**) Abacus plot of hourly detections and the regions each unique shark present at Padaro Beach before the upwelling event was detected. Regions are ordered by latitude.

**Figure 7:**
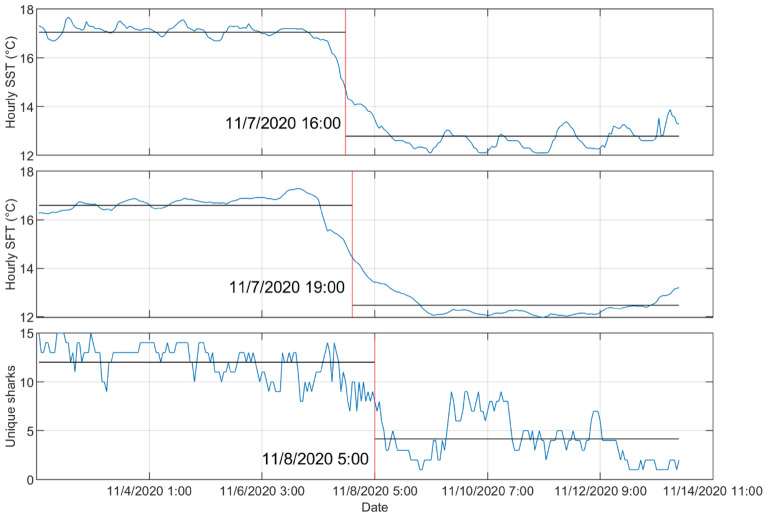
Change point analysis from hourly averaged data from 1 to 16 November of sea surface temperature, sea floor temperature, and the number of unique sharks present. The change points occurred on 7 November at 16:00 PST, 7 November at 19:00 PST, and 8 November at 05:00 PST respectively as shown by the solid red lines.

Analysis at higher temporal resolution (hourly) of the time around when the sharks left the area (1 November–16 November) showed changes in behavior not seen at the daily time scale (Fig. 6). All tagged sharks left Padaro Beach between 8–9 November with 11 out of 16 sharks traveling at least 32 km south before returning to Padaro on the same day. Change point analysis showed that there was a ~ 10–13 h difference in when the mean temperature dropped and when many JWS started their initial response to exit the area (Fig. 7).

## Discussion

Coastal water temperatures in Southern California routinely fluctuate 2 °C within a 12-h period, can have an average at least a 10 °C (SST) change throughout the year, and can also be subject to periods of increased magnitude often due to storms and upwelling events^44,45^. Sinnett & Feddersen (2014) found that nearshore water temperatures may drop 4 °C in 36 min and rapid oscillations of 0.5 °C in 90 s for 1–2 hours can occur^46^, while an increase of 8 °C in nearshore waters in less than two months was reported during the 2018 Southern California marine heatwave^47^. As JWS in this environment experience natural thermal variability which oscillates over the course of the day, month, and season, the rate and magnitude of these changes may influence an individual’s decision to remain within or depart from a specific location. JWS may therefore seek out locations that offer the most suitable environmental conditions within their thermal preferenda, leading to aggregating behavior as well as influencing emigration from an aggregation site.

From 15 May to 7 November before the sharks left the aggregation site (receiver array), daily average water temperatures fluctuated from 16.1 to 23.8 °C (sea surface) and 12.7–20.2 °C (seafloor). The time periods of these fluctuations prior to 7 November, when the daily average seafloor temperature fell below 14 °C, lasted from 1 to 5 days. Although 14 °C is believed to be the lower behavioral threshold of JWS thermal preferenda^27^, the water column remained largely stratified during these periods, with surface temperatures remaining ~ 2 °C warmer than seafloor temperatures on average, thus providing sharks with vertical thermal options within the same general area (Fig. 2a). Vertical movements indicate that sharks may be using different thermal habitats during different times of day, moving into warmer surface waters during the day and cooler, deeper waters during crepuscular periods^33^. The change point analyses indicated that by 8 November the sharks began a mass emigration from this aggregation site when the ambient water temperature dropped below 14 °C uniformly throughout the water column and across the entire site and remained so for eight days. This abrupt temperature change was a result of a strong coastal upwelling event lasting from 6 November (index = 238) to 8 November (index = 189).

Prior to the upwelling event in November, the change point analysis showed the first partial emigration event occurred when the water temperature rose, and the thermocline dissipated around 19 August. Despite new individuals being tagged around that time, three days later ~ 15% of the tagged sharks at the aggregation site began to leave throughout the next ~ 10 days. While there was no downwelling event documented for that area on 19 August (Bakun index = 53), the thermocline dissipated and water temperatures across the entire site and water column increased. Although this partial emigration pattern could have occurred due to several factors: (e.g., late summer movements between Southern California aggregations sites^34^, emigrating/departing sharks may have responded to the water becoming homogenously warmer with no option of thermal refugia (cooler water) across the water column. Although these sharks are regional endotherms, capable of maintaining core body temperatures above that of the external environment, the inability to regulate body temperature through the selection of temperature profiles across the water column over an extended period may render the habitat unfavorable. Thus, the combination of ambient water temperature rise, and the breakdown of the thermocline likely initiated an emigration event in a portion of the resident population of JWS.

When looking at the daily resolution of shark presence, tagged sharks were observed to leave the array within five days of the onset of a sub-optimal temperature regime. Upon exploring the data at an hourly resolution, it was observed that initial shark mass emigration began 10–12 h following a sharp temperature decrease to below the 14 °C thermal threshold, beginning on 7 November. Before this event, tagged sharks within the study area routinely experienced a 2 °C temperature fluctuation within a 12-h period due to daily tidal cycles and warming from solar radiation. JWS within coastal/nearshore aggregation sites may therefore become habituated to relatively moderate temperature oscillations within a 10–12 h period. Sharks may also compensate for such oscillations by seeking out preferable thermal strata within the water column^33^. Individuals may therefore tolerate the physiological costs of a greater fluctuation in temperature, as such costs may be outweighed by the benefits of adequate protection and food availability^21^.

However, with the onset of the strong upwelling event in early November, the water column within the study area fell dramatically, and became largely homogeneous, with little to no difference seen in temperatures at the surface and seafloor (Fig. 2). Thus, the entire water column fell below a thermal threshold of 14°C^27^, without any available thermal strata where sharks might find temporary refuge. The thermal environment likely became intolerable to many of the individuals, which triggered the mass emigration event seen in the ~ 5-day period following. Eleven of the 16 sharks that dispersed in this period were subsequently detected at receiver stations in Ventura County (< 35 km away) (Fig. 6), before returning to the study site, then leaving once again. One possible explanation for this observation is that sharks left the study site to seek out a habitat with more tolerable/preferable temperatures, then returned briefly to the study area when this search was unsuccessful, as nearby water temperatures were similar to those at the study site (See Fig. S1, Supporting Information), before leaving once more as thermal conditions within the array had not improved. Following the mass emigration, several individuals were detected at the Northern Channel Islands, which at that time had warmer seafloor temperatures than Padaro Beach, remaining above 14 °C (See Fig. S1, Supporting Information). That phenomenon is unusual as the Channel Islands are typically exposed to cooler water than inshore habitats^48^.

Three animals remained within the study area during the November strong upwelling event, all of which represented the three smallest size classes tagged. Although these smaller sharks may be more thermally sensitive, they did not leave the study area in response to the abrupt change in temperature, as was seen for all other tagged sharks that were resident within the study area at the time. Larger (older) sharks may therefore have knowledge and experience of alternative locations to seek out when the thermal environment becomes intolerable. Physiological costs associated with relocation may be greater than those of remaining in place^33^ and are accompanied by the uncertainty of adequate resources (e.g., food, protection). Thus, these younger, more naïve individuals may have remained in place rather than leave due lack of knowledge of alternative locations with potentially more suitable conditions. Ultimately, this temperature event lasted for ~ ten days before warming back up to temperatures over 14 °C. All but the three smallest sharks left the study area for the rest of the year.

Previous reports of migration events documenting movements of juvenile white sharks have relied on broader –spatial-scale thermal data (e.g., satellite SST or NOAA buoy data) to quantify seasonally related movements. Weng et al. tracked six JWS for 24–182 days with pop-off archival tags and found that the four young-of-the-year individuals (147–156 cm) migrated south into Mexico in the winter when the water in California dropped in temperature, while the two three-year-old individuals (248–250 cm) stayed in Southern or Central California^19^. These findings were supported by White et al., who developed a habitat suitability model showing that Southern California was suitable in temperature for six to eight months out of the year, based on both SPOT and miniPAT tracking data from 10 JWS between 2010 and 2013^27^. Of the tagged animals that departed the study site during the November upwelling event, only one individual, a larger juvenile (250–275 cm TL), migrated south as opposed to moving between different areas. This observation is contrary to those documented in previous studies and may be a function of climate anomalies that affect the marine environment, leading to habitat expansion and changes in seasonal habitat use patterns.

With climate change continuing to alter historic temperature patterns in Southern California, it is possible JWS may forego their typical migration patterns in favor of remaining in the nearshore waters of Southern California regardless of their size. If individuals continue to show high site fidelity to nursery aggregation sites, their temperature selectivity may lower as they increase in mass due to their increased thermal inertia, increasing their thermal tolerance to lower temperatures. This could lead to individuals remaining in the habitat despite temperature cues to migrate, so long as abundant and suitable prey persist. Alternatively, much as we saw in 2020, larger juveniles may continue to be the first to leave as temperatures become suboptimal as they may be more experienced and/or willing to leave an aggregation site in search of more productive areas. Further research is needed to elucidate the propensity of different size classes to leave their residency habitat in response to changing temperatures.

Animals may emigrate from an area for reasons other than thermal changes. For instance, dramatic weather events are known to induce mass emigration where tagged sharks have been shown to leave areas just prior to storms with barometric pressure drops being the migratory cue^49^. In addition to abiotic factors, lack of sufficient prey^7^ or increased predator risk^50^ may also drive the need for an animal to move. The pattern of residency (e.g., the number of individuals coming to and leaving the aggregation site) may be more indicative of resource-related limitations for individuals. It is possible the emigration of JWS from the aggregation site was attributed to mass emigration of prey responding to the environmental cue. However, small elasmobranchs, which are thought to be important prey of JWS^51^, may be more temperature sensitive as they are ectotherms and therefore more likely to be easily caught by JWS which thermo-energetic advantage^21^. If environmental cues are not enough to trigger a mass emigration and individuals stay in the area, the top-down impact on prey populations is unknown but may in turn result in a breakdown of the aggregation^34,52^. From an ecological perspective, the largest impacts resulting from JWS remaining within nursery aggregation locations year-round may be realized as trophic pressures within ecological communities associated with the habitat. In addition, the reduction or cessation of seasonal migration in resident JWS would result in an increase in larger-sized individuals using those areas over time. Thus, sustained pressure from feeding/foraging combined with ontogenetic shifts in diet^52,53^ would likely put additional new pressures on the wider community^54^.

This study provides methods for quantifying the environmental thermal thresholds and the timing of which may drive mass emigration in a large, highly mobile, species. Understanding this cue may help predict changes in migratory patterns under current changing climate conditions. Such understanding is important to informing best practice in marine and coastal management and conservation initiatives and public safety.

## Supplementary Information

Supplementary Information.

## Data Availability

Raw data supporting the conclusions of this article will be made available by the corresponding authors upon request.
