# Supplementary material for: Quantifying thermal cues that initiate mass emigrations in juvenile white sharks
Source: Sci Rep. 2022 Nov 18;12:19874. doi: 10.1038/s41598-022-24377-1 (PMC9674695; doi:10.1038/s41598-022-24377-1)
Supplement: Supplementary file 1 — Supplementary Information. [file 41598_2022_24377_MOESM1_ESM.pdf]

Supporting Information

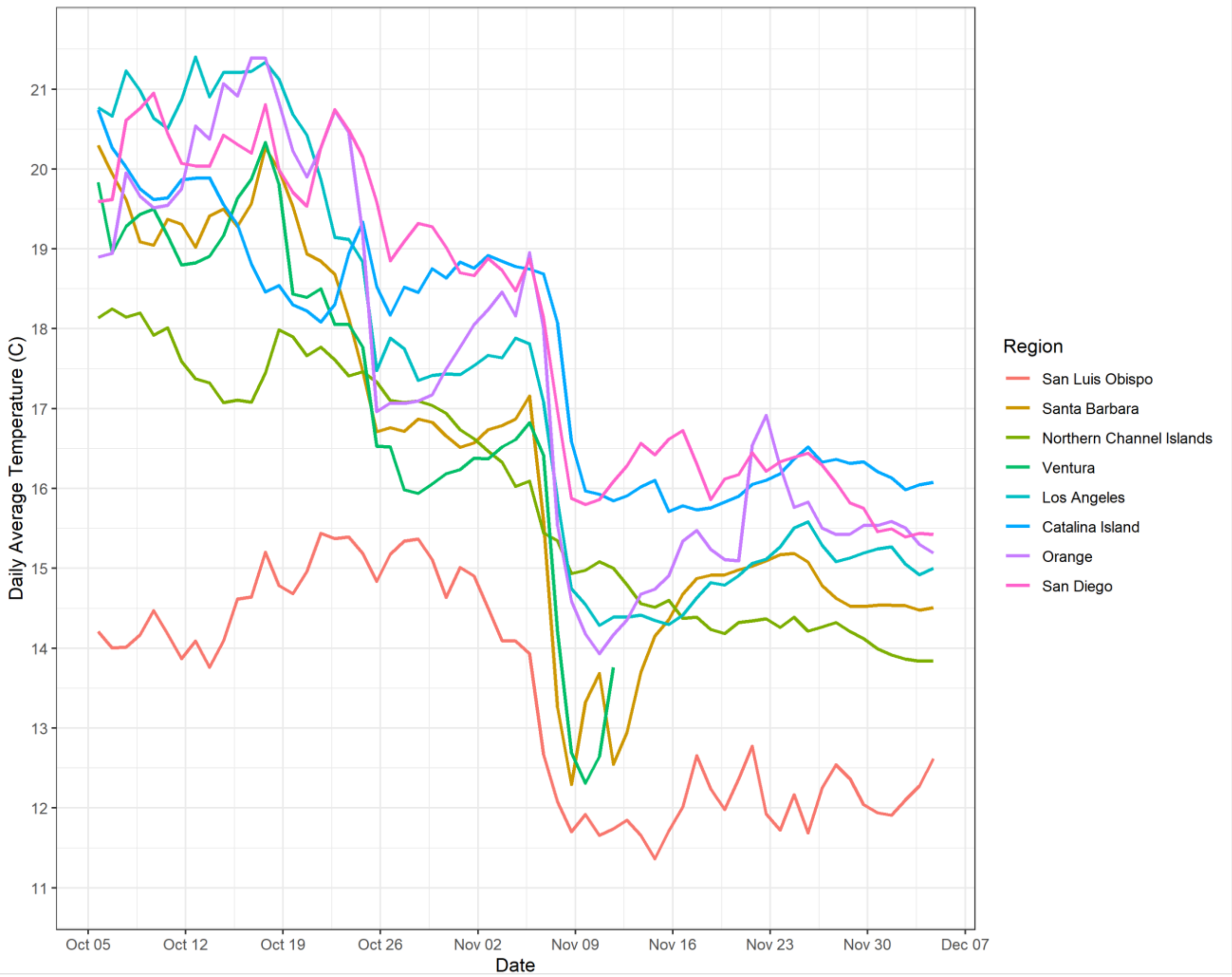

Figure S1. Daily average sea floor temperature as recorded hourly by the network of acoustic receivers placed along the Central to Southern California Coast. Averages are separated by regions of coast as well as ordered by latitude.
